# Predictors of Cognitive Decline in Individuals with Subjective Cognitive Decline of the DELCODE Study Cohort

**DOI:** 10.1002/alz70857_105570

**Published:** 2025-12-24

**Authors:** Lena Sannemann, Katharina Buerger, Julian Hellmann‐Regen, Luca Kleineidam, Christoph Laske, Robert Perneczky, Oliver Peters, Josef Priller, Alfredo Ramirez, Anja Schneider, Annika Spottke, Matthis Synofzik, Stefan J. Teipel, Michael Wagner, Jens Wiltfang, Renat Yakupov, Emrah Düzel, Frank Jessen, the DELCODE Study Group

**Affiliations:** ^1^ University of Cologne, Faculty of Medicine and University Hospital Cologne, Department of Psychiatry, Cologne, Germany; ^2^ Institute for Stroke and Dementia Research (ISD), University Hospital, LMU, Munich, Germany; ^3^ German Center for Neurodegenerative Diseases (DZNE), Munich, Germany; ^4^ German Center for Neurodegenerative Diseases (DZNE), Berlin, Germany; ^5^ Charité – Universitätsmedizin Berlin, corporate member of Freie Universität Berlin and Humboldt‐Universität zu Berlin – Institute of Psychiatry and Psychotherapy, Berlin, Germany; ^6^ ECRC Experimental and Clinical Research Center, Charité Universitätsmedizin Berlin, Berlin, Germany; ^7^ Department of Old Age Psychiatry and Cognitive Disorders, University Hospital Bonn and University of Bonn, Bonn, Germany; ^8^ German Center for Neurodegenerative Diseases (DZNE), Bonn, Germany; ^9^ German Center for Neurodegenerative Diseases (DZNE), Tuebingen, Germany; ^10^ Section for Dementia Research, Hertie Institute for Clinical Brain Research and Department of Psychiatry and Psychotherapy, University of Tuebingen, Tuebingen, Germany; ^11^ Ageing Epidemiology (AGE) Research Unit, School of Public Health, Imperial College London, London, United Kingdom; ^12^ Munich Cluster for Systems Neurology (SyNergy), Munich, Germany; ^13^ Department of Psychiatry and Psychotherapy, University Hospital, LMU Munich, Munich, Germany; ^14^ Department of Psychiatry and Psychotherapy, School of Medicine and Health, Technical University of Munich, and German Center for Mental Health (DZPG), Munich, Germany; ^15^ University of Edinburgh and UK DRI, Edinburgh, United Kingdom; ^16^ Department of Psychiatry and Psychotherapy, Charité, Charitéplatz 1, Berlin, Germany; ^17^ Division of Neurogenetics and Molecular Psychiatry, Department of Psychiatry and Psychotherapy, Faculty of Medicine and University Hospital Cologne, University of Cologne, Cologne, Germany; ^18^ Department of Psychiatry and Glenn Biggs Institute for Alzheimer's and Neurodegenerative Diseases, San Antonio, TX, USA; ^19^ Excellence Cluster on Cellular Stress Responses in Aging‐Associated Diseases (CECAD), University of Cologne, Cologne, Germany; ^20^ Department of Neurology, University of Bonn, Bonn, Germany; ^21^ German Center for Neurodegenerative Diseases (DZNE), Tübingen, Germany; ^22^ Division of Translational Genomics of Neurodegenerative Diseases, Hertie Institute for Clinical Brain Research and Center of Neurology, University of Tübingen, Tübingen, Germany; ^23^ Department of Psychosomatic Medicine, Rostock University Medical Center, Rostock, Germany; ^24^ German Center for Neurodegenerative Diseases (DZNE), Rostock, Germany; ^25^ Department for Cognitive Disorders and Old Age Psychiatry, University Hospital Bonn, Bonn, Germany; ^26^ Department of Psychiatry and Psychotherapy, University Medical Center Goettingen, University of Goettingen, Goettingen, Germany; ^27^ Neurosciences and Signaling Group, Institute of Biomedicine (iBiMED), Department of Medical Sciences, University of Aveiro, Aveiro, Portugal; ^28^ German Center for Neurodegenerative Diseases (DZNE), Goettingen, Germany; ^29^ German Center for Neurodegenerative Diseases (DZNE), Magdeburg, Germany; ^30^ Institute of Cognitive Neurology and Dementia Research (IKND), Otto‐von‐Guericke University, Magdeburg, Germany; ^31^ Institute of Cognitive Neurology and Dementia Research (IKND), Otto‐von‐Guericke University, Magdeburg, Sachsen Anhalt, Germany

## Abstract

**Background:**

Subjective cognitive decline (SCD) refers to a self‐perceived, persistent cognitive decline compared to previous levels in individuals with objectively unimpaired cognition. Studies have repeatedly shown associations of SCD characteristics with amyloid pathology and increased risk of future cognitive decline, especially in memory‐clinic settings. The aim of this project is to model individual differences of cognitive decline in individuals with SCD.

**Method:**

Latent Growth Curve Model (LGCM) analysis was applied to individuals with SCD from the DZNE Longitudinal Cognitive Impairment and Dementia (DELCODE) study. We chose a sample of *n* = 203 participants who showed a decline on the Preclinical Alzheimer's Cognitive Composite (PACC5) score over five years. First, a two‐factor linear growth model was fitted on the annualized PACC5 data. We then calculated and compared two models to which we added the following baseline predictors: 1) plasma Aß42/40, plasma ptau181, ApoE‐4‐carrier status and hippocampal volume (biological model), and 2) Geriatric Depression Scale (GDS), Geriatric Anxiety Inventory–Short Form (GAIS‐SF) and Neuropsychiatric Inventory Questionnaire (NPI‐Q) total scores (neuropsychiatric model).

**Result:**

The LGCM of longitudinal PACC‐5 scores yielded adequate model fit for a linear model (*X* ^2^(16)=67.5, *p* < .001, CFI = 0.93, SMRM=0.07, AIC=1437.10). The baseline PACC score was ‐0.03 (*SE* = 0.05, *p* = .533) and average cognition declined slightly over time by ‐0.13 (*SE* = 0.01, *p* < .001). The biological model showed an improvement in fit, with an AIC of 742.30. Here, we observed a positive relationship between plasma Aß42/40 and the intercept (*B* = 7.60, *SE* = 3.01, *p* = .012) and a negative relationship between plasma ptau181 and the intercept (*B* = ‐0.22, *SE* = 0.09, *p* = .016). Plasma Aß42/40 was the only significant predictor of the PACC5 slope in this model (*B* = 1.35, *SE* = 0.62, *p* = .030). In comparison, the AIC value for the neuropsychiatric model was 1341.17, with the GDS total score being negatively related to the PACC5 slope (*B* = ‐0.03, *SE* = 0.01, *p* = .009).

**Conclusion:**

These results add to gaining a better understanding of SCD trajectories and specific predictors of cognitive decline, which is relevant to power future clinical trials in this population.

